# Cerebrospinal fluid features in comatose survivors of cardiac arrest: A pilot study

**DOI:** 10.1371/journal.pone.0270954

**Published:** 2022-07-26

**Authors:** Marine Paul, Sarah Benghanem, Sybille Merceron, Hugo Bellut, Florence Dumas, Amandine Henry, Fabrice Bruneel, Jean-Pierre Bedos, Alain Cariou, Stéphane Legriel

**Affiliations:** 1 Intensive Care Unit, Centre Hospitalier de Versailles—Site André Mignot, Le Chesnay, France; 2 AfterROSC Study Group, Paris, France; 3 Intensive Care Unit, Cochin Hospital, Paris, France; 4 Sorbonne Paris Cité-Medical School, Paris Descartes University, Paris, France; 5 Emergency Department, Cochin Hospital, Paris, France; 6 Université de Paris, PARCC, INSERM, Paris, France; 7 Paris Sudden-Death-Expertise-Centre, Paris, France; 8 Microbiology Department, Centre Hospitalier de Versailles—Site André Mignot, Le Chesnay, France; 9 University Paris-Saclay, UVSQ, INSERM, CESP, PsyDev Team, Villejuif, France; Fondazione IRCCS Policlinico San Matteo, ITALY

## Abstract

**Introduction:**

Lumbar puncture is among the investigations used to identify various neurological conditions, including some that can cause cardiac arrest (CA). However, CA per se may alter cerebrospinal fluid (CSF) characteristics. Few studies have investigated CSF findings after CA. In this descriptive work, we assessed the frequency and risk factors of abnormal CSF findings after CA and the contribution of CSF analysis to the etiological diagnosis.

**Materials and methods:**

We retrospectively studied data from prospectively established databases of consecutive patients who were admitted to two French ICUs in 2007–2016 with sustained return of spontaneous circulation (ROSC) after CA and who underwent lumbar puncture as an etiological investigation.

**Results:**

Of 1984 patients with sustained ROSC, 55 (2.7%) underwent lumbar puncture and were included. Lumbar puncture identified a neurological cause of CA in 2/55 (3.6%) patients. Nonspecific CSF abnormalities were noted in 37/53 (69.8%) patients. By multivariate analysis, postresuscitation shock was positively associated with CSF abnormalities (OR, 6.92; 95% confidence interval [95%CI], 1.62–37.26; *P* = 0.013). A no-flow time above 6 minutes (OR, 0.19; 95%CI, 0.03–1.11; *P* = 0.076) and a respiratory cause of CA (OR, 2.91; 95%CI, 0.53–23.15; *P* = 0.24) were not statistically associated with CSF abnormalities. Nonspecific CSF abnormalities were not significantly associated with poor outcomes (Cerebral Performance Category ≥3; *P* = 0.06).

**Conclusions:**

Lumbar puncture, although infrequently performed, may contribute to the etiological diagnosis of CA, albeit rarely. Nonspecific CSF abnormalities seem common after CA, notably with postresuscitation shock, and may be related to blood-brain barrier disruption. These findings may help to interpret CSF findings after CA. Further studies are warranted to assess our results.

## Introduction

Cardiac arrest (CA) is among the most common causes of death in Europe and the United States despite advances in resuscitation and intensive care [1]. During the early phase after the return of spontaneous circulation (ROSC), identifying the cause is crucial, both to allow specific treatments that may improve patient outcomes and to lower the risk of recurrent CA. Recent guidelines recommend considering coronary angiography and cerebral and/or chest computed tomography (CT), depending on the CA circumstances and the electrocardiographic findings after ROSC [2]. Unfortunately, even when these guidelines are applied, over 40% of patients receive no definitive etiologic diagnosis and may therefore be at higher risk for delayed or inappropriate treatments and for poorer outcomes [3].

According to recent data, 7% of CAs are due to neurological causes [4]. Cerebral CT is the first-line investigation when a neurological cause is suspected. However, the analysis of cerebrospinal fluid (CSF) obtained by lumbar puncture (LP) may be useful also. Although several studies have assessed the usefulness of various CSF biomarkers for neuroprognostication [5, 6], the possible contribution of CSF analysis to the etiological diagnosis of CA has not been investigated, and neither have the characteristics of CSF after CA been described. In addition to neurological causes of CA, a potential source of CSF abnormalities is blood-brain barrier (BBB) disruption due to anoxia [7]. Improved knowledge of CSF characteristics after CA due to neurological and other causes would help to interpret CSF findings.

We therefore designed a descriptive, retrospective study of prospectively established databases to evaluate the diagnostic contribution of CSF findings and to describe the frequency and risk factors of CSF abnormalities in patients admitted to the ICU with sustained ROSC after CA.

## Materials and methods

We used two prospectively collected databases established at the Cochin hospital and Versailles hospital (#NCT03594318), respectively. Both are CA referral centers that serve the southern and southwest areas of the Paris conurbation (France). Data collection was approved by the ethics committee of the French Intensive Care Society (#CESRLF_12–384 and 20–41), and the data were collected anonymously in compliance with French data protection legislation (French Data Protection Authority #MR004_2209691) [8, 9]. Verbal informed consent obtained from each surrogate and each patient in case of awakening was recorded in the medical file. The study is reported according to the STROBE statement.

### Study setting and early patient management

In France, when the emergency services receive a call reporting a suspected case of out-of-hospital CA, the fire department and mobile emergency unit system dispatch a team to the scene. The staff in each mobile emergency unit includes at least one physician trained in emergency medicine in compliance with international guidelines [10], who delivers advanced life support. Patients with in-hospital CA are initially managed by the nurses and/or bedside physician until the arrival of an emergency physician, intensivist, or anesthesiologist, who performs advanced life support. Patients with a stable return of spontaneous circulation (ROSC) are admitted to the intensive care unit (ICU).

### Postresuscitation diagnostic evaluation

As recommended in current guidelines [2], a standardized diagnostic workup is started immediately to allow the prompt identification and treatment of the cause of CA. In patients with clinical and/or electrocardiographic evidence of myocardial ischemia and in those with no obvious non-cardiac cause of CA, coronary angiography is performed at hospital arrival, before ICU admission. If prodromal symptoms or the clinical findings suggest a respiratory or neurological cause of CA, CT of the chest or brain, respectively, may be chosen as the best first-line investigation. When the first-line investigation fails to detect a cause, further tests are considered [11]. Additionally, after ICU admission, laboratory tests are performed routinely to look for metabolic abnormalities or toxic substances, as dictated by the clinical history. LP for CSF collection is performed in patients with meningeal syndrome and when deemed appropriate by the physician in charge. All these investigations were available in both participating centers 24 h a day and 7 days a week.

### Study population

All eligible patients entered into the Cochin and Versailles CA databases between January 2007 and December 2016 were included if they were older than 18 years, had stable ROSC at hospital admission, and underwent LP as part of the etiological workup. We did not include patients who underwent LP for other reasons or who had a traumatic LP defined as a CSF white cell count/red cell count <1/1000 [12]. We also excluded patients with postmortem LP.

### Study objectives

The primary objective was to assess the potential contribution of CSF analysis to identification of the cause of CA. The secondary objectives were to describe the frequency of CSF abnormalities (defined as protein >0.45 g/L and/or white cell count >5/mm^3^) and to identify factors associated with CSF abnormalities in patients whose CSF analysis did not contribute to the etiological diagnosis. We also evaluated potential associations linking CSF abnormalities to survival and functional outcome at ICU discharge [13].

### Data collection

Demographic data and data related to the CA were collected prospectively in the two electronic databases according to the Utstein style [14]. These data included age and sex, place of CA occurrence, initial rhythm, no-flow and low-flow times, presence of a witness, bystander CPR, number of defibrillations, and epinephrine use. We also recorded comorbidities, initial ST-segment elevation, coronary angiography and/or CT findings, and definitive cause of CA. The following were collected in the ICU: use of targeted temperature management, presence of postresuscitation shock, postanoxic status epilepticus, and/or awakening defined as a response to commands with a motor Glasgow Coma Scale score of 6.

To further investigate the value of CSF analysis after CA, we used standardized forms to retrospectively collect the following data from the prehospital and ICU records: symptoms preceding CA including headache, focal signs, confusion, coma, and seizures (but not meningeal stiffness); sepsis before LP; CSF characteristics (biochemistry, cytology, and culture results), time from CA to CSF collection, blood sample findings on the day of LP, and CSF/serum protein quotient.

The functional outcome was assessed using the Cerebral Performance Category (CPC) at ICU discharge, and causes of death were recorded [15–17]. We defined a favorable outcome as a CPC score of 1 or 2 at ICU discharge.

### Statistical analysis

Quantitative parameters were described as median (interquartile range [IQR]) or mean (range) and qualitative parameters as number (percentage). We compared categorical variables using Fisher’s exact test and continuous variables using the Wilcoxon rank-sum test.

We first described CA characteristics in the patient groups defined by whether CSF analysis contributed to identify the cause of CA. We then described the CSF characteristics in the group whose CSF analysis did not contribute to identify the cause. To assess risk factors for CSF abnormalities after CA, we compared patients with vs. without CSF abnormalities, first by univariate analysis then by building a multivariate model using variables associated with *P* values that were lower than 0.05 or were clinically relevant. Continuous variables were transformed into dummy variables. We applied the Hosmer-Lemeshow goodness-of-fit test and used the C-statistic to estimate the area under the receiver operating characteristics (ROC) curve. Finally, we looked for associations linking CSF abnormalities to survival and functional outcome at ICU discharge.

All tests were two-sided and *p* values <0.05 were considered significant. Analyses were performed using R statistical software version 3.1.2 (R Foundation for Statistical Computing, Vienna, Austria, http://www.R-project.org, accessed November 12, 2020).

## Results

**Fig 1:**
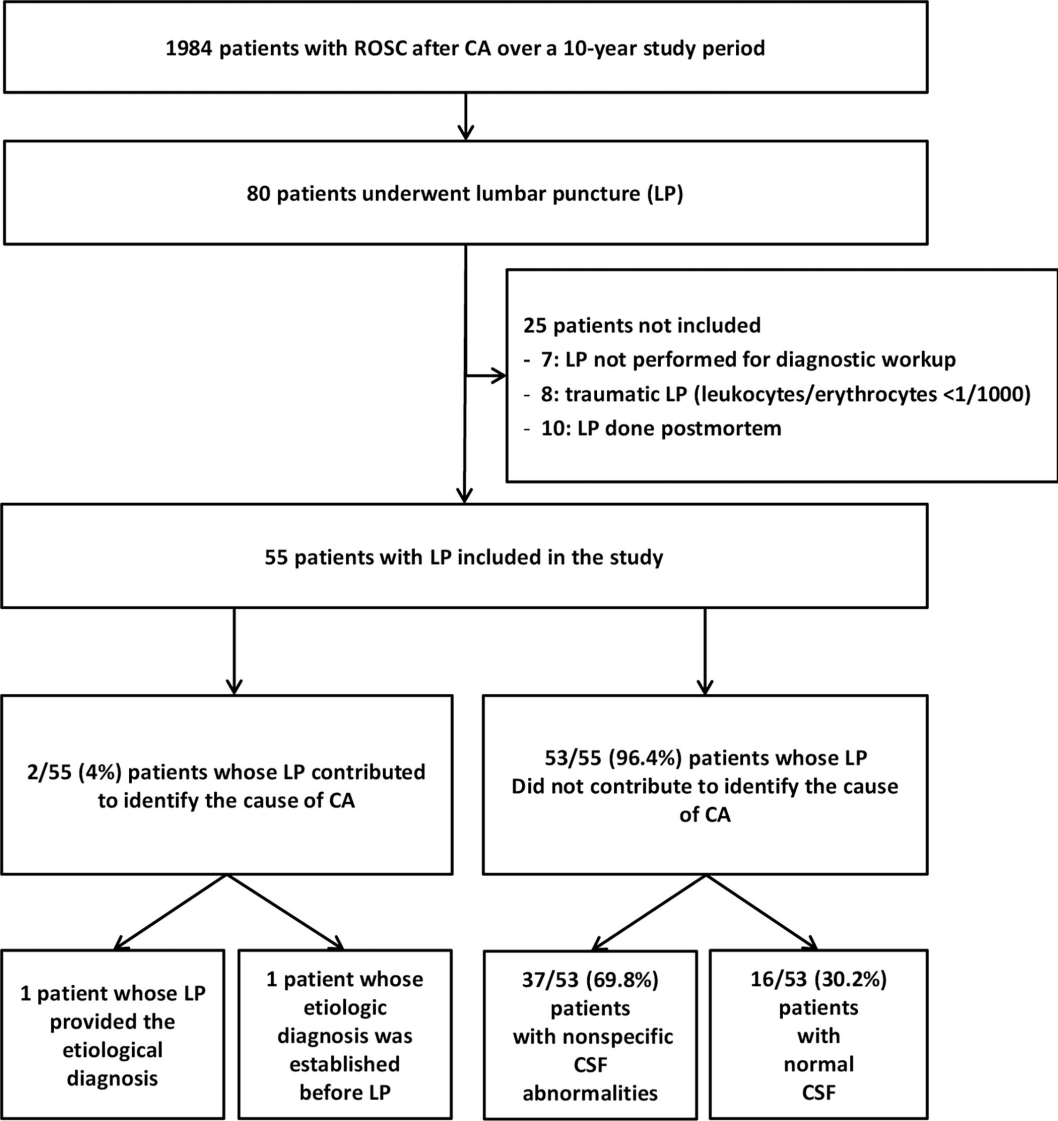
Patient flow diagram. ROSC: Return of spontaneous circulation; CA: Cardiac arrest; CT: Computed tomography.

Fig 1 is the patient flow chart. Of the 1984 patients admitted with stable ROSC after CA, 55 (2.7%) underwent LP at a median of 1 day [1–2] after CA and were included in the study.

### Characteristics and diagnostic workup

Table 1 and S1 Table in S1 File report the patient characteristics and diagnostic investigations performed to identify the cause of CA. S1 Fig in S1 File shows the first-line, second-line, and third-line investigations. Overall, cerebral CT was done in 47 (85.5%) patients, cerebral MRI in 5 (9%) patients, coronary angiography in 28 (51%) patients, and chest CT in 29 (53%) patients. The cause of CA was identified in 45 (82%) of the 55 patients and was respiratory in 18 (33%), neurologic in 12 (22%), cardiac in 6 (11%), metabolic in 7 (13%), and septic in 2 (3.6%).

**Table 1:** Diagnostic workup, identified causes, and outcomes in 55 patients who underwent lumbar puncture after cardiac arrest.

	N (%) or Median [interquartile range]		
	All patients	CSF analysis contributive	CSF analysis not contributive
	n = 55 (100%)	n = 2/55 (3.6%)	n = 53/55 (96.4%)
**Prodromal signs**			
Neurological signs/symptoms before CA	41 (74.5)	1 (50.0)	38 (71.7)
Confusion to coma	16 (29.1)	1 (50.0)	15 (28.3)
Seizure	19 (34.5)	1 (50.0)	18 (33.9)
Neurological focal signs	5 (9.1)	1 (50.0)	4 (7.5)
Headache	1 (1.8)	0	1 (1.9)
**Tests for cause of CA other than lumbar puncture**			
ST elevation by ECG	6 (11.1)	0	6 (11.5)
Coronary angiography	28 (50.9)	1 (50.0)	27 (50.9)
Cerebral CT	47 (85.5)	2 (100)	45 (84.9)
Cerebral MRI	5 (9.1)	1 (50.0)	5 (9.4)
Chest CT	29 (52.7)	0	29 (54.7)
**Lumbar puncture**			
First-line	7 (12.7)	0	7 (13.2)
Second-line	23 (41.8)	1 (50.0)	22 (41.5)
Third-line	25 (45.5)	1 (50.0)	24 (45.2)
Time from CA to LP, days	1 [1–2]	1.5 [1.2–1.7]	1 [1–2]
**Cause of CA**			
Respiratory	18 (32.7)	0	18 (33.9)
Neurologic	12 (21.8)	2 (100)	10 (18.8)
Cardiac	6 (10.9)	0	6 (11.3)
Metabolic	7 (12.7)	0	7 (13.2)
Septic shock	2 (3.6)	0	2 (3.7)
Undetermined	10 (18.2)	0	10 (18.8)
**Outcomes**			
ICU length of stay, days	6.0 [3.0–9.0]	5.5 [4.2–6.7]	6.0 [3.0–9.0]
Awakening during ICU stay	20 (36.4)	0	20 (37.7)
CPC score at ICU discharge			
1–2	19 (34.5)	0	19 (35.8)
3–4	0	0	0
5	36 (65.5)	2	34 (64.2)
Reason for ICU death (n = 36)			
Multiorgan failure	9 (16.4)	0	9 (16.9)
Anoxic encephalopathy	20 (36.4)	2 (100)	20 (37.7)
Brain death	6 (10.9)	0	4 (7.5)
Other	1 (1.8)	0	1 (1.8)

LP: lumbar puncture; CA: cardiac arrest; ECG: electrocardiogram; CT: computed tomography; MRI: magnetic resonance imaging; ICU: intensive care unit; CPC: Cerebral Performance Category

### Contribution of cerebrospinal fluid (CSF) analysis to the aetiologic diagnosis

LP was assessed in cases of suspicion of a neurological cause for cardiac arrest in 74.5% of patients. CSF analysis helped to identify a neurological cause of CA in 2/55 (3.6%) patients; of these 2 patients, 1 had encephalitis (direct contribution) and the other subarachnoid hemorrhage with possible confusion on the cerebral CT-scan between blood and contrast from previous coronary angiography.

### Patients with nonspecific cerebrospinal fluid (CSF) abnormalities

Among the 53 patients whose CSF analysis did not help to identify the cause of CA, 37 (69.8%) had CSF abnormalities (Table 2). CSF protein elevation was found in 34 (94.4%) of 36 patients, whereas CSF white-cell-count elevation was far less common, with only 10 (27%) of the 37 patients. The highest CSF white-cell count in patients with nonspecific CSF abnormalities was 144/mm^3^. CSF lactate was above >2.4mmol/L in 10 (91%) of 11 patients.

**Table 2:** Cerebrospinal fluid characteristics in the 53 patients whose cerebrospinal fluid (CSF) analysis did not contribute to identify the cause of cardiac arrest.

		n (%) or Median (interquartile range) / Mean [range]		
		CSF did not contribute to identify the cause of cardiac arrest		
	All patients	Patients with normal CSF	Patients with abnormal CSF^b^	*P* value
	n = 53	n = 16/53 (30.2%)	n = 37/53 (69.8%)	
CSF white-cell count, per mm^3^	1 (0–3) / 7.10 [0–144]	0 (0–1.3) / 0.87 [0–4]	2 (0–5) / 10 [0–144]	0.02
CSF white-cell count >4/mm^3^	10 (18.9)	0	10 (27.1)	0.023
CSF neutrophil count, per mm^3^ ^a^	1 (1–2)	1 (1–1)	1 (1–5)	-
CSF lymphocyte count, per mm^3^ ^a^	0 (0–2.2)	0 (0–0)	0 (0–3)	-
CSF red-cell count, per mm^3^	14 (1–216)	7.5 (1–9.75)	20 (1–221)	0.45
CSF protein, g/L	0.54 (0.41–0.65) / 0.68 [0.2–3.99]	0.4 (0–0.4) /0.35 [0.2–0.42]	0.6 (0.5–0.8) / 0.8 [0.4–3.9]	<0.0001
CSF protein >0.45 g/L	34/52 (65.4)	0	34/36 (94.4)	<0.001
CSF glucose, mmol//L	4.7 (4.1–6.0)	4.8 (4.5–5.2]	4.8 (4.0–6.0)	0.99
CSF lactate, mmol/L	4.6 (3.4–7.7)	2.6 (2.3–5.5)	4.7 (4.3–7.7)	0.35
Blood protein, g/L	64 (53–68)	67 (65–68)	60 (52–68)	0.06
Blood glucose, mmol/L	7.0 (5.9–9.2)	6.95 [6.45;8.675]	7.3 (5.9–11.8)	0.98
CSF/serum protein quotient	0.009 (0.006–0.01)	0.005 (0.004–0.006)	0.01 (0.009–0.01)	0.03
CSF/serum glucose quotient	0.6 (0.5–0.8)	0.6 [0.575;0.8]	0.7 (0.5–0.8)	0.93
Positive CSF culture	0	0	0	-
Abnormal cells	0	0	0	-

CSF: cerebrospinal fluid

^a^in patients with CSF white-cell count >4/mm^3^

^b^Abnormal CSF was defined as CSF white-cell count >4/mm^3^ and/or CSF protein >0.45 g/L.

### Factors associated with nonspecific cerebrospinal fluid (CSF) abnormalities

Table 3 compares the patients with versus without nonspecific CSF abnormalities. By multivariate analysis, postresuscitation shock was positively associated with abnormal CSF (OR, 6.92; 95%CI, 1.62–37.26; *P* = 0.013). A no-flow time longer than 6 minutes (OR, 0.19; 95%CI, 0.03–1.11; *P* = 0.076) and a respiratory cause of CA (OR, 2.91; 95%CI, 0.53–23.15; *P* = 0.24 were not statistically associated with abnormal CSF. The *P* value of the Hosmer-Lemeshow test for this multivariate analysis was 0.69 with an area under the ROC curve of 0.79. Patients with postresuscitation shock had a higher CSF/serum protein quotient with a median of 0.010 [0.478–0.011] vs. 0.007 [0.005–0.001] in patients without shock, P = 0.002.

**Table 3:** Demographic and cardiac arrest characteristics in patients whose cerebrospinal fluid analysis did not contribute to identify the cause of cardiac arrest (n = 53).

	N (%) or Median [interquartile range]		
	Normal CSF	Abnormal CSF^a^	*P* value
	n = 16/53 (30.2%)	n = 37/53 (69.8%)	
**Demographic characteristics and comorbidities**			
Age, years	49 [39–65]	56 [40–74]	0.24
Males	9 (56.3)	26 (70.3)	0.36
Diabetes mellitus	17 (18.7)	6 (16.2)	1.00
Spinal cord compression	1 (6.3)	1 (2.7)	0.52
Hematological malignancy	0	2 (5.4)	–
Epilepsy	4 (25.0)	5 (13.5)	0.43
**Cardiac arrest characteristics**			
Neurological signs/symptoms before CA	10 (62.5)	21 (56.8)	0.34
Confusion to coma	2 (12.5)	13 (35.1)	0.11
Seizure	8 (50.0)	10 (27.0)	0.13
Focal neurologic signs	1 (6.3)	3 (8.1)	1.00
Headache	0	1 (2.7)	–
Cardiac arrest in a public place	3 (18.7)	5 (13.5)	0.69
Arrest witnessed/monitored	13 (81.2)	32 (86.5)	0.69
Bystander CPR	12 (75.0)	31 (83.8)	0.47
Shockable rhythm	5 (31.2)	6 (16.2)	0.27
Total number of defibrillations before ROSC	0 (0–2)	0 (0–1)	0.16
Use of epinephrine	11 (68.8)	30 (81.1)	0.48
Total epinephrine dose before ROSC, mg	1 [0–3]	2 [1–4]	0.081
Time from collapse to CPR (no-flow), min	4 [2–10]	0 [0–3]	0.004
Time from CPR to ROSC (low-flow), min	16 [9–21]	10 [6–20]	0.41
**Cause of CA**			0.15
Respiratory	2 (12.5)	16 (43.2)	
Neurologic	5 (31.3)	5 (13.5)	
Cardiac	2 (12.5)	4 (10.8)	
Other	7 (43.7)	12 (32.5)	
Lactate concentration on ICU admission, mmol/L	3.9 [2.5–7.4]	6.5 [2.3–11.0]	0.40
Edema on cerebral CT	1/14 (7.1)	7/31 (22.6)	0.30
Time from cardiac arrest to LP, days	1 [1–2]	1 [1–2]	0.51
Targeted temperature management (32–36°C) on day 1	14 (87.5)	31(83.8)	1.00
Sepsis before LP	1 (6.3)	9 (24.3)	0.25
Postresuscitation shock	5 (31.2)	26 (70.3)	0.01
Renal replacement therapy	4 (25.0)	9 (24.3)	1.00
Status epilepticus before LP	2 (66.7)	3 (42.9)	1.00

LP: lumbar puncture; CA: cardiac arrest; CT: computed tomography; MRI: magnetic resonance imaging; ICU: intensive care unit; ROSC: return of spontaneous circulation

^a^Abnormal CSF was defined as CSF white-cell count >4/mm^3^ and/or CSF protein >0.45 g/L.

### Patient outcomes

Overall ICU mortality was 65% (36/55). Table 1 shows the causes of death. Both patients whose CSF analysis contributed to the etiological diagnosis died. Nonspecific CSF abnormalities were more common in patients with poor outcomes defined as CPC 3, 4, or 5 (73% versus 27% of those with CPC 1 or 2), although the difference was not statistically significant (*p* = 0.06). S2 Table in S1 File reports the CSF features in patients with favorable versus unfavorable outcomes. The only factor significantly associated with outcome was the CSF/serum protein quotient (*p* = 0.017), with higher values in patients who experienced worse outcomes.

## Discussion

CSF analysis was performed in only 2.7% of patients admitted to the ICU with stable ROSC after CA and contributed to the etiological diagnosis in only 2 (3.6%) patients. Among patients whose CSF analysis did not contribute to the etiological diagnosis, over two-thirds had nonspecific CSF abnormalities, among which the most common was protein elevation. Postresuscitation shock was an independent and significant risk factor for abnormal CSF. The CSF/serum protein quotient was significantly associated with a poor outcome.

LP was performed only very rarely in our population. Few previously published data are available with which to compare our results. Most studies of CSF analysis after CA focused on the neuroprognostication accuracy of CSF biomarkers reflecting neuronal damage [5, 6, 18]. We are not aware of previous studies investigating the potential contribution of CSF analysis to the etiological diagnosis or the presence of CSF abnormalities unrelated to the etiology. In previous studies, CSF analysis was performed in 5.3% of patients with neurological causes of CA and stable ROSC at hospital admission, chiefly as part of the etiological workup [4], and in 40% of patients with CA complicating convulsive status epilepticus [4, 19]. Given, the small proportion of noncardiac causes of CA, recent guidelines focus on the indications of coronary angiography, cerebral CT, and chest CT. Important factors are the patient’s medical history; the presence of cardiac, respiratory, or neurological prodromal symptoms; the circumstances of CA onset; and the physical findings at the scene. In practice, LP is not a first-line investigation unless there is evidence of a neurological cause whose identification may be helped by CSF analysis. Obstacles to LP include anticoagulant and/or antiplatelet treatments, although in a recent study the risk of spinal hematoma after LP was the same in patients with and without coagulopathy [20]. Another concern is cerebral herniation, and cerebral CT may be required before LP is performed. As expected, LP was mainly performed as a second- or third-line investigation in our study, predominantly in patients with neurological prodromal symptoms before CA. Work is clearly needed to determine the indications of LP after CA. An optimal etiological workup is crucial to determine when specific treatments are appropriate and available to improve patient outcomes. In previous studies, ICU survival was higher when the etiology was identified [3, 21]. In addition, identifying the cause may allow measures to minimize the risk of recurrent CA. Finally, knowledge of the causes of CA is important from a public health perspective. LP identified the cause of CA in 3.6% of our patients.

Over two-thirds of our patients without identified neurological causes of CA had nonspecific CSF abnormalities, of which the most common was protein elevation (94%), followed by white-cell count elevation (27%), with the highest recorded value being white-cells 144/mm^3^. Knowledge of the range of CSF abnormalities seen after CA may help to interpret CSF results. Indeed, CSF cell count above the range reported here should prompt further investigations for a neurological cause of CA. Although several studies assessed the usefulness of various CSF biomarkers for neuroprognostication [5, 6], none described the CSF characteristics. Direct brain injury may cause nonspecific CSF abnormalities, as described in stroke and status epilepticus and CSF findings may be difficult to interpret in patients with brain injury [22].

All the hypotheses put forward to explain these findings focus on the blood-brain barrier (BBB), which is composed of cerebral endothelial cells, a capillary basement membrane, pericytes, and astrocytes. The BBB regulates the penetration of plasma constituents into the cerebral parenchyma [23, 24]. In healthy individuals, most of the proteins found in the CSF come from the serum, although some are synthesized by the choroid plexus or within the brain. The passage of serum proteins into the CSF varies with the status of the BBB [23]. Normal BBB permeability is defined as a CSF/serum albumin quotient <0.007 [25, 26]. The ischemic/reperfusion injuries that result from CA may induce CSF abnormalities via several mechanisms.

First, CSF changes may be due to systemic inflammation. Elevated levels of proinflammatory cytokines have been found in the CSF after CA [27, 28]. In our study, postresuscitation shock was independently associated with nonspecific CSF abnormalities. Postresuscitation shock results from the cytokine storm and free-radical release induced by ischemia/reperfusion during and after CA [29]. These abnormalities may cause BBB alterations similar to those described in acute sepsis and cirrhosis including changes in tight junctions, damage to endothelial cells, activation of astrocytes and microglia, alterations of multiple transport pathways and receptors, and penetration of peripheral immune cells [30]. In our study, the CSF/serum protein quotient was higher in patients with vs. without postresuscitation shock.

Second, CSF changes may occur in response to primary brain injury with anoxic neuronal damage. Cerebral anoxia may lead not only to BBB disruption but also to the production of specific cerebral proteins, resulting in CSF abnormalities. High-mobility group box 1 (HMGB1) released by necrotic brain cells may act as an early inflammation trigger inducing the local recruitment of proinflammatory cytokines, independently of BBB alterations [6]. Increases in neuronal specific enolase, protein S100B, T-tau protein, and neurofilament have also been reported after CA [6, 31]. These compounds are markers for brain injury. We initially expected that greater severity of brain injury would be associated with CSF abnormalities. However, a no-flow time above 6 minutes was not associated with abnormal CSF. The no-flow and low-flow times were reported retrospectively by the medical team that delivered on-scene resuscitation. These times are often approximative and go unrecorded in some cases. In our study, of the 6 patients with no information on the no-flow time, 4 (10.8%) were among the 37 with abnormal CSF and 2 (12.5%) among the 16 with normal CSF. Moreover, the study population was highly selected and therefore not fully representative of all patients with CA. Indeed, our population had a short median no-flow time of 0 min (0–4.5). Also, cerebral hypoxia before CA due to respiratory causes was not associated with abnormal CSF. Furthermore, of the 45 patients who had an initial cerebral CT scan, 8 had cerebral edema, including 1 (7.1%) of 14 patients with normal CSF and 7 (22.6%) of 31 patients with abnormal CSF (*P* = 0.4 by univariate analysis). Further studies in a larger population are needed to clarify the links between cerebral anoxia and CSF characteristics. Finally, we found no associations linking CSF abnormalities to sepsis before LP, spinal-cord compression, diabetes, hematological malignancy, or epilepsy before LP.

ICU mortality was 65% in the overall population of patients with LP after CA. Both patients whose CSF analysis provided etiological information died. Identifying a neurological cause of CA has been reported to carry a very poor prognosis [4, 9]. In our study, ICU mortality in the patients whose CSF analysis did not contribute to the diagnosis but showed nonspecific abnormalities was 64%. A higher CSF/serum protein quotient was the only variable significantly associated with a poor outcome. Similarly, a prospective study in 21 patients found that the CSF/serum albumin quotient was higher in the subgroup of 10 patients with poor outcomes than in the other patients [32]. These findings support the existence of BBB disruption after CA. Finally, in our cohort, 56% of deaths among patients with nonspecific CSF abnormalities occurred after life-support was withdrawn due to severe postanoxic encephalopathy.

Our study has several limitations. First, given the retrospective design and small sample size, the extent to which our findings apply to the full spectrum of patients with CA is unclear. We included consecutive patients with LP after ICU admission with stable ROSC after CA. However, LP was not performed routinely in patients meeting predefined criteria and was contraindicated in many patients due to anticoagulant exposure, notably due to previous angiography. Moreover, both participating ICUs were high-volume centers whose recruitment may not reflect that of ICUs overall. Second, we considered only LPs done at the early phase after CA, as part of the emergent etiological workup. Delayed CSF analysis may provide important information. One study found that the CSF/serum albumin quotient increased between 24 h and 72 h after ROCS, and others evidenced an increase in CSF protein levels after 2–3 weeks [7]. However, our focus was on the potential usefulness of CSF analysis for the etiological diagnosis and on the frequency and risk factors of nonspecific CSF abnormalities after CA. Finally, the unavailability of data on BBB function obtained from the CSF/blood albumin ratio and specific MRI sequences precluded an assessment of the potential influence of systemic and neurological inflammation on CSF protein levels [25, 33]. Moreover, we did not adjust the CSF protein values for age [34].

## Conclusion

In conclusion, although rarely performed after CA, LP may contribute to the identification of a neurological cause of CA. In our study, CSF analysis as a second-line investigation identified a neurological cause in 3.7% of patients. Nonspecific CSF abnormalities were common after CA with postresuscitation shock, perhaps due to BBB disruption. These findings may help clinicians to interpret CSF findings after CA. Further studies are warranted to clarify the links between CA and CSF abnormalities.

## Supporting information

S1 File (DOCX)

## Abbreviations

BBB: blood-brain barrier
CA: cardiac arrest
CPC: Cerebral Performance Category
CPR: cardiopulmonary resuscitation
CSF: cerebrospinal fluid
CT: computed tomography
EEG: electroencephalogram
ICU: intensive care unit
IQR: interquartile range
ROSC: return of spontaneous circulation
