# Supplementary material for: Cerebrospinal fluid features in comatose survivors of cardiac arrest: A pilot study
Source: PLoS One. 2022 Jul 26;17(7):e0270954. doi: 10.1371/journal.pone.0270954 (PMC9321437; doi:10.1371/journal.pone.0270954)
Supplement: S1 File — (DOCX) [file pone.0270954.s001.docx]

**Table S1: Main characteristics of the 55 patients who underwent lumbar puncture after cardiac arrest**

|  | N (%) or median (interquartile range) |
| --- | --- |
|  | n = 55 |
| **Cardiac arrest Utstein criteria** |  |
| Age, years | 53 (40-67) |
| Male sex | 37 (67.3) |
| Cardiac arrest in a public place | 9 (16.4) |
| Out of hospital cardiac arrest |  |
| Arrest witnessed/monitored | 47 (85.5) |
| Bystander CPR | 44 (80%) |
| Shockable first recorded rhythm | 12 (21.8) |
| Total number of defibrillations before ROSC | 0 (0-1) |
| Use of epinephrine | 43 (78.2) |
| Total epinephrine dose before ROSC, mg | 2 (1-4) |
| Time from collapse to CPR (no-flow), min | 0 (0-4.5) |
| Time from to ROSC (low-flow), min | 15 (7-20) |
| Lactate concentration on ICU admission, mmol/L | 5.8 (2.3-10.7) |
| Targeted temperature management (32-36°C) on day 1 | 47 (85.5) |
| Post-resuscitation shock | 33 (60) |
| Renal replacement therapy | 13 (23.6) |

CPR, cardiopulmonary resuscitation; ROSC, return of spontaneous circulation; ICU, intensive care unit

**Figure S1: Diagnostic work-up in 55 patients with a lumbar puncture after cardiac arrest**

**
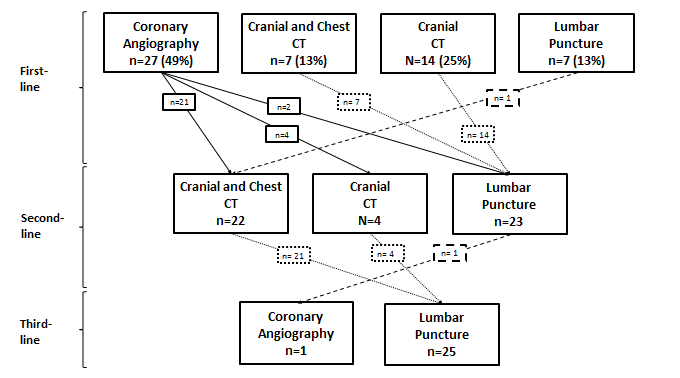
**

**Table S2: Comparison of patients with favourable versus unfavourable outcomes in the subgroup whose cerebrospinal fluid analysis did not contribute to the aetiologic diagnosis n=53**

|  | **N (%) or Median (Interquartile Range**) | | |
| --- | --- | --- | --- |
|  | **CPC 1-2**  **n=19/53 (35.8%)** | **CPC 3-4-5**  **n=34/53 (64.2%)** | ***p* value** |
| Age, years | 53 (36-73) | 55 (41-66) | 0.99 |
| Male sex | 12 (63.2) | 23 (67.7) | 0.77 |
| Neurological signs/symptoms before CA |  |  |  |
| Confusion to coma | 31.6 | 26.5 | 0.76 |
| Seizure | 31.6 | 35.29 | 1.00 |
| Neurological focal signs | 5.3 | 8.82 | 1.00 |
| Headache | 5.3 | 0 | 0.36 |
| Abnormal CSF | 10 (52.6) | 27 (79.4) | 0.062 |
| CSF red-cell count, per mm^3^ | 10 (1-218.5) | 14.5 (1-207.5) | 0.87 |
| CSF white-cell count, per mm^3^ | 1 (0-2) | 2 (0-3.75) | 0.13 |
| CSF neutrophil count, per mm^3^* | 1 (1-1) | 1 (1-4) | - |
| CSF lymphocyte count, per mm^3^* | 0 (0-0) | 0 (0-9) | - |
| CSF protein, g/L | 0.43 (0.38-0.63) | 0.57 (0.49-0.65) | 0.082 |
| CSF glucose, mmol//L | 4.8 (4.3-5.9) | 4.7 (4.2-6) | 0.98 |
| CSF lactate, mmol/L | 3.7 (3.2-4) | 5.5 (4.4-9) | 0.14 |
| CSF/serum protein quotient | 0.008 (0.006-0.009) | 0.01(0.008-0.011) | 0.017 |
| CSF/serum glucose quotient | 0.6 (0.5-0.7) | 0.7 (0.5;0.8) | 0.30 |

CPC: Cerebral Performance Category; CA: cardiac arrest; CSF: cerebrospinal fluid

*in patients with CSF white cell count >4/mm3

**CHECK LIST STROBE**

|  | **Item No.** | **Recommendation** | **Page No.** |
| --- | --- | --- | --- |
| **Title and abstract** | 1 | (*a*) Indicate the study’s design with a commonly used term in the title or the abstract | 4 |
|  |  | (*b*) Provide in the abstract an informative and balanced summary of what was done and what was found | 4 |
| **Introduction** |  |  |  |
| Background/rationale | 2 | Explain the scientific background and rationale for the investigation being reported | 5 |
| Objectives | 3 | State specific objectives, including any prespecified hypotheses | 5 |
| **Methods** |  |  |  |
| Study design | 4 | Present key elements of study design early in the paper | 6-7 |
| Setting | 5 | Describe the setting, locations, and relevant dates, including periods of recruitment, exposure, follow-up, and data collection | 6-7 |
| Participants | 6 | (*a*) *Cohort study*—Give the eligibility criteria, and the sources and methods of selection of participants. Describe methods of follow-up  *Case-control study*—Give the eligibility criteria, and the sources and methods of case ascertainment and control selection. Give the rationale for the choice of cases and controls *Cross-sectional study*—Give the eligibility criteria, and the sources and methods of selection of  participants | 7 |
|  |  | (*b*) *Cohort study*—For matched studies, give matching criteria and number of exposed and unexposed  *Case-control study*—For matched studies, give matching criteria and the number of controls per  case |  |
| Variables | 7 | Clearly define all outcomes, exposures, predictors, potential confounders, and effect modifiers.  Give diagnostic criteria, if applicable | 7 |
| Data sources/ measurement | 8* | For each variable of interest, give sources of data and details of methods of assessment (measurement). Describe comparability of assessment methods if there is more than one group | 8 |
| Bias | 9 | Describe any efforts to address potential sources of bias | 8 |
| Study size | 10 | Explain how the study size was arrived at |  |
| Quantitative variables | 11 | Explain how quantitative variables were handled in the analyses. If applicable, describe which groupings were chosen and why | 9 |
| Statistical methods | 12 | (*a*) Describe all statistical methods, including those used to control for confounding | 9 |
|  |  | (*b*) Describe any methods used to examine subgroups and interactions | 9 |
|  |  | (*c*) Explain how missing data were addressed |  |
|  |  | (*d*) *Cohort study*—If applicable, explain how loss to follow-up was addressed  *Case-control study*—If applicable, explain how matching of cases and controls was addressed *Cross-sectional study*—If applicable, describe analytical methods taking account of sampling strategy |  |
|  |  | (*e*) Describe any sensitivity analyses |  |
| **Results** |  |  |  |
| Participants | 13* | (a) Report numbers of individuals at each stage of study—eg numbers potentially eligible, examined for eligibility, confirmed eligible, included in the study, completing follow-up, and analysed | 9 |
|  |  | (b) Give reasons for non-participation at each stage |  |
|  |  | (c) Consider use of a flow diagram | 9 |
| Descriptive data | 14* | (a) Give characteristics of study participants (eg demographic, clinical, social) and information on exposures and potential confounders | 9 |
|  |  | (b) Indicate number of participants with missing data for each variable of interest |  |
|  |  | (c) *Cohort study*—Summarise follow-up time (eg, average and total amount) |  |
| Outcome data | 15* | *Cohort study*—Report numbers of outcome events or summary measures over time | 9-10 |
|  |  | *Case-control study—*Report numbers in each exposure category, or summary measures of exposure |  |
|  |  | *Cross-sectional study—*Report numbers of outcome events or summary measures |  |
| Main results | 16 | (*a*) Give unadjusted estimates and, if applicable, confounder-adjusted estimates and their precision (eg, 95% confidence interval). Make clear which confounders were adjusted for and why they were included | 9-10 |
|  |  | (*b*) Report category boundaries when continuous variables were categorized |  |
|  |  | (*c*) If relevant, consider translating estimates of relative risk into absolute risk for a meaningful time  period |  |
| Other analyses | 17 | Report other analyses done—eg analyses of subgroups and interactions, and sensitivity analyses | 9-10 |
| **Discussion** |  |  |  |
| Key results | 18 | Summarise key results with reference to study objectives | 11 |
| Limitations | 19 | Discuss limitations of the study, taking into account sources of potential bias or imprecision. Discuss both direction and magnitude of any potential bias | 15 |
| Interpretation | 20 | Give a cautious overall interpretation of results considering objectives, limitations, multiplicity of  analyses, results from similar studies, and other relevant evidence | 15 |
| Generalisability | 21 | Discuss the generalisability (external validity) of the study results | 16 |
| **Other information** |  |  |  |
| Funding | 22 | Give the source of funding and the role of the funders for the present study and, if applicable, for the original study on which the present article is based | NA |
|  |  |  |  |
